# Curative treatment for oligometastatic gastroesophageal cancer– results of a prospective multicenter study

**DOI:** 10.1007/s00423-024-03575-7

**Published:** 2024-12-16

**Authors:** N. Norén, I. Rouvelas, L. Lundell, M. Nilsson, B. Sunde, E. Szabo, D. Edholm, J. Hedberg, U. Smedh, M. Hermansson, M. Lindblad, F. Klevebro

**Affiliations:** 1 Department of Surgery and Oncology, CLINTEC, Karolinska Institutet, Dep. of Upper Gastrointestinal Diseases, Karolinska University Hospital, Stockholm, Sweden; 2 Örebro University Hospital, Örebro, Sweden; 3 Department of Surgery, Biomedical and Clinical Sciences, Linköping University, Linköping, Sweden; 4 Department of Surgical Sciences, Uppsala University, Uppsala University Hospital, Uppsala, Sweden; 5 Sahlgrenska University Hospital, Gothenburg, Sweden; 6 Department of Surgery, Skåne University Hospital and Department of Clinical Sciences, Lund University, Lund, Sweden; 7 Department of Surgery, Odense University Hospital, Odense, Denmark

**Keywords:** Oligometastatic gastroesophageal cancer, Gastric cancer, Esophageal cancer, Postoperative complications, Overall survival

## Abstract

**Purpose:**

Oligometastatic gastroesophageal cancer is a clinical entity with no standard treatment recommendation. Treatment with curative intent has recently emerged as an option for selected patients in contrast to the traditional palliative treatment strategy. This prospective study aimed to assess the safety and efficacy of combined systemic and local treatment with curative intent for patients with oligometastatic gastroesophageal cancer.

**Methods:**

In a multicenter study, consecutive patients with gastroesophageal cancer and metastases in the liver and/or extra-regional lymph nodes were screened for inclusion. Eligible patients were offered curatively intended perioperative chemotherapy followed by surgical resection or liver ablation. Primary endpoints were treatment safety and feasibility. Secondary outcomes included postoperative mortality, treatment response, progression-free survival, and overall survival. Subgroup analyses were stratified based on oligometastatic location.

**Results:**

A total of 29 (82.9%) patients completed treatment with surgical resection (93.1%), liver ablation (3.4%), or definitive chemoradiotherapy (3.4%). Postoperative complications were found in 19 (73.1%) patients, whereas postoperative mortality was 0%. The most common complications included infection (34.6%) and respiratory complications (34.6%). Median overall survival was 20.9 months (interquartile range 11.2–42.6) from diagnosis and 17.0 months (interquartile range 6.4–35.9) from surgery in patients who were treated with neoadjuvant chemotherapy followed by surgery. Median progression-free survival was 5.8 months (interquartile range 3.1–11.3).

**Conclusion:**

This study found curative treatment to be a relatively safe option, with an overall survival of 20.8 months and no postoperative mortality.

**Supplementary Information:**

The online version contains supplementary material available at 10.1007/s00423-024-03575-7.

## Introduction

Gastric and esophageal cancer are the third and sixth leading causes of cancer-related mortality, accounting for over 1 million deaths globally every year [1]. Despite improved treatment during the last decade, the overall prognosis remains poor [2]. This is mainly a result of the large proportion of patients presenting with disseminated disease at diagnosis and the high recurrence rate after curative treatment [2–5]. Metastases commonly manifest in the liver or the extra-regional lymph nodes in esophageal cancer and as extra-regional lymph node metastases or peritoneal metastases in gastric cancer [6–8]. At this stage, treatment has traditionally been given with palliative intent, resulting in a survival of less than 12 months [4, 6, 9, 10].

Recent publications have addressed the question of whether surgical resection has a role in the treatment of metastatic gastroesophageal cancer. Emerging evidence indicates that patients with a limited metastatic burden, known as oligometastatic disease, could under certain circumstances be offered treatment with curative intent [11–16]. The concept of oligometastatic disease was introduced in 1995 by Hellman and Weichselbaum and has been increasingly implemented over the past few years [17]. A clear definition of oligometastatic disease in gastroesophageal cancer has not been available until recently, and previous studies tended to include heterogeneous patient groups. In 2022, oligometastatic disease was defined as up to three distant metastases in a single organ or involvement of one extra-regional lymph node station [14, 17, 18].

Surgical resection of oligometastatic disease is well-established in colorectal cancer. It has demonstrated improved overall survival and progression-free survival in breast, prostate, and small-cell-lung cancer [19, 20]. A standard treatment approach for oligometastatic disease in gastroesophageal cancer is currently lacking due to the absence of completed randomized controlled trials. However, there is an ongoing debate regarding patient selection for curative treatment since previous studies indicate improved overall survival for selected patients compared with palliative treatment [11–14, 21–24].

## Methods

### Study design

**Fig. 1:**
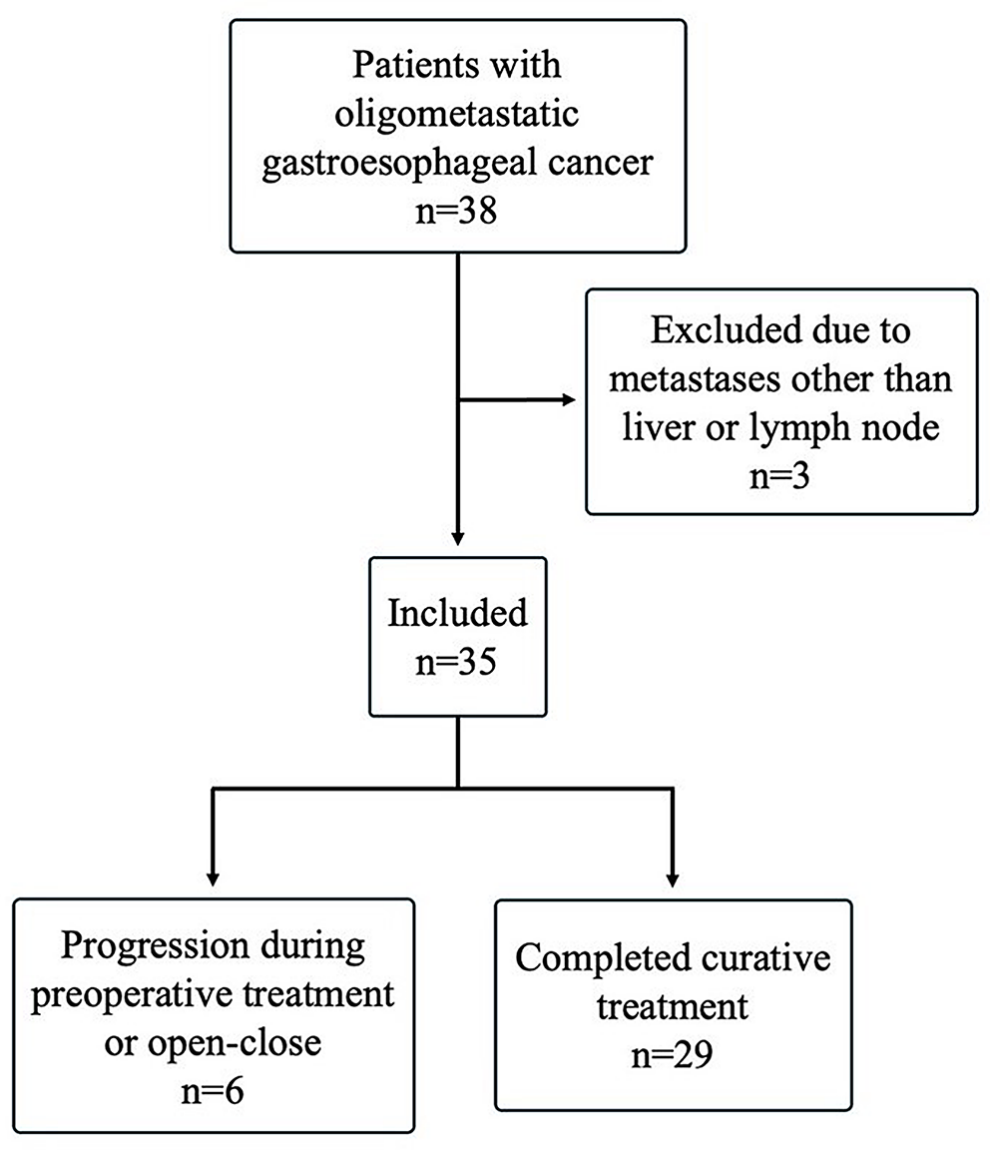
Patients considered for inclusion at the regional tumor boards between 2017 and 2022

This was a prospective multicenter cohort study. Consecutive patients with histologically verified esophageal, junctional, or gastric adenocarcinoma and clinical staging, including gastroscopy and fluorodeoxyglucose positron emission tomography combined with 3-phase computer tomography (PET-CT) scan, were screened for inclusion at the regional tumor boards between 2017 and 2022. Patients with oligometastatic disease in the liver and/or cervical, thoracic, or abdominal extra-regional lymph nodes who met the inclusion criteria were included (Fig. 1).

#### Inclusion criteria

Metastatic disease was diagnosed either at the time of or within 12 months of the initial diagnosis of the primary tumor (i.e., synchronous) or after 12 months of the initial diagnosis of the primary tumor (i.e., metachronous). Oligometastatic disease in the liver was defined as 5 or fewer uni- or bilobular metastases with the largest metastasis being up to 50 mm on magnetic resonance imaging (MRI) with liver-specific contrast. Extra-regional lymph node stations considered as oligometastatic disease included station 104, 16a2, and 16b1 for esophageal adenocarcinoma and stations 101, 104, 106, 16a2, and 16b1 for gastric adenocarcinoma. An Eastern Cooperative Oncology Group (ECOG) performance status of 2 or less, being at least 18 years old, sufficient kidney and liver function, and no comorbidity that could significantly impact overall survival were all mandatory for consideration. Junctional tumors classified as Siewert type I and II were categorized as esophageal cancer, while Siewert type III was categorized as gastric cancer. Written informed consent was required from all participants. The regional medical records were used to obtain clinical data, including patient characteristics, treatment-related data, and survival.

#### Exclusion criteria

Patients with more than 5 liver metastases, liver metastases larger than 50 mm, or distant metastases outside the liver or the above-stated lymph node stations were excluded. In addition, patients considered to have unresectable tumors or not considered fit for surgery (e.g., general condition or comorbidity) were also excluded.

### Treatment

Before initiating treatment, all patients were assessed in a multidisciplinary tumor board meeting at each participating center. Systemic chemotherapy, according to local preference, was given for 6–12 weeks. Adverse events were graded according to the Common Terminology Criteria for Adverse Events (CTCAE version 6.0) classification based on detailed descriptions in the medical records. The tumor response to preoperative chemotherapy was evaluated based on radiological assessment at the preoperative multidisciplinary tumor board meeting. In the absence of tumor progression at re-staging, patients were re-assessed for surgical resection. Surgical resection was scheduled 3–6 weeks after the last chemotherapy course and included esophagectomy, total gastrectomy, or subtotal gastrectomy based on tumor location for the primary tumor. The metastases were treated with lymph node dissection, liver resection, or liver ablation based on the clinician’s choice at the multidisciplinary tumor board meeting. In case of synchronous oligometastatic disease, the primary tumor and metastatic lesion were treated simultaneously. In case of metachronous oligometastatic disease, the treatment only included the metastases as the primary tumor had been treated previously.

### Postoperative treatment and follow-up

Patients could receive 3 months of postoperative chemotherapy according to the recommendation from the postoperative multidisciplinary tumor board. Clinical controls were performed at 1, 3, and 6 months and then every 6th month. CT scans were performed every 6th month for five years or on suspicion of recurrent disease.

The patients who did not respond to systemic chemotherapy or were considered unresectable at re-staging received palliative treatment according to the local standard of care, which could include palliative chemotherapy, palliative radiotherapy, targeted treatments, or best supportive care. These patients were followed up according to local recommendations.

### Outcomes and sample size calculations

The primary outcome of the study was the safety and feasibility of combined systemic and local treatment with curative intent for patients with oligometastatic gastroesophageal cancer. Enrollment was planned to continue until the patients had completed three months of chemotherapy and had been re-evaluated for resection. A resection rate of more than 60% after initial chemotherapy was considered clinically acceptable. Assuming a significance level of 0.05 (α = 0.05) and a power of 90% (β = 0.10), it was calculated that 26 patients should be included in the first part of the study. Thus, if 15 or fewer out of the first 26 consecutive patients had been resected, the hypothesis would be rejected, and the study would be closed after the first stage of accrual. If 16 or more patients had been resected, an additional 19 patients would be accrued in the second stage, in total 45 patients.

Secondary outcomes included postoperative complications according to the Clavien-Dindo classification [25], rate of radical resections, progression-free survival, overall survival, treatment response based on tumor regression grade according to Becker [26], and patient-reported quality of life according to European Organisation for Research and Treatment of Cancer (EORTC) core questionnaire QLQ-C30 (version 3.0) and the disease-specific module for esophageal cancer QLQ-OES25 [27, 28]. The response to the questionnaire was reported as mean and standard deviation (SD). A higher score indicated more severe symptoms in the symptom subscale of QLQ-C30 and QLQ-OES25 and a better function in the functional subscale of QLQ-C30. Tumor regression grade for the metastases was calculated with the same grading system as for the primary tumors. Human epidermal growth factor receptor 2 (HER2) status was assessed by immunohistochemistry as part of the routine tumor assessment. Progression-free survival was calculated from the date of operation to recurrence. Treatment outcomes, including postoperative complications, rate of radical resection, and quality of life, were based on the patients who completed curative treatment (i.e., resection, liver ablation, or definitive chemoradiotherapy, *n* = 29). The survival analysis was based on the patients who underwent surgery or liver ablation and were treated for adenocarcinoma (*n* = 25) to represent a more homogeneous cohort. Patients who received palliative treatment due to progression during preoperative chemotherapy, had squamous cell carcinoma, or received definitive chemotherapy were not included in the survival analysis.

### Statistical analysis

Categorical variables were reported as absolute numbers and percentages. Normally distributed continuous variables were reported as mean and SD, and non-normally distributed variables as median and interquartile range (IQR). Kaplan-Meier graphs were used to demonstrate overall and progression-free survival. A Cox proportional hazard model was applied to calculate the hazard ratio (HR) and compare subgroups with 95% confidence intervals (CI). The significance level was set at < 0.05. Statistical analyses were performed using R version 2024.04.1 + 748 (2024-04-01). Ethical approval for the study was obtained from the Stockholm Regional Ethics Committee 2015/1985-31/3 and 2018/2496-32.

## Results

The resection rate among the first 26 patients was 81% and the study continued. Due to slow accrual, inclusion was prematurely closed at 35 patients in order to analyze the results. The histological tumor subtype was adenocarcinoma in 32 (91.4%) patients (Table 1). Three patients (8.6%) had squamous cell carcinoma and were included by mistake (i.e., protocol violation). They were reported regarding baseline characteristics, surgical technique, and postoperative complications but were not included in the survival analysis. Among the 35 enrolled patients, 28 (80%) completed curatively intended treatment including preoperative chemotherapy followed by metastasectomy or liver ablation. Twenty-two (78.6%) of them had simultaneous resection of the primary tumor. One (2.9%) patient received definitive chemoradiotherapy (as an alternative curative option) after an attempt at surgical resection that was interrupted due to tumor progression. Similar to the patients with squamous cell carcinoma, this patient was not included in the survival analysis. Six (17.1%) patients did not complete the planned curatively intended treatment due to tumor progression during preoperative chemotherapy or non-resectable disease diagnosed at exploration. These patients received palliative treatment instead (Table 1, Supplementary Information).

**Table 1:** Patient and tumor characteristics at baseline

*n*/total (%)	Total	Completed Curative treatment	Curative resection Adenocarcinoma
**No. of patient**	*N* = 35	*N* = 29	*N* = 25
Karolinska University Hospital	29/35 (82.9)	23/29 (79.3)	20/25 (80.0)
Other Hospitals	6/35 (17.1)	6/29 (20.7)	5/25 (20.0)
**Age** median (IQR)	66.0 (61.0-70.5)	64.0 (60.0–68.0)	64.0 (60.0–68.0)
**Sex**			
Female	12/35 (34.3)	11/29 (37.9)	8/25 (32.0)
Male	23/35 (65.7)	18/29 (62.1)	17/25 (68.0)
**Charlson Comorbidity Index** mean (SD)	2.6 (± 1.3)	2.4 (± 1.2)	2.4 (± 1.2)
**Body Mass Index** mean (SD)	24.5 (± 4.3)	24.6 (± 4.6)	25.0 (± 4.5)
**Performance status**			
ECOG 0	20/34 (58.8)	14/28 (50.0)	11/24 (45.8)
ECOG 1	14/34 (41.2)	14/28 (50.0)	13/24 (54.2)
Missing	1 (2.9)	1 (3.4)	1 (4.0)
**Primary tumor location**			
Esophagus	2/35 (5.7)	2/29 (6.9)	1/25 (4.0)
Siewert I-II	26/35 (74.3)	24/29 (82.8)	21/25 (84.0)
Stomach	7/35 (20.0)	3/29 (10.3)	3/25 (12.0)
**Histology**			
Adenocarcinoma	32/35 (91.4)	26/29 (89.7)	25/25 (100)
Squamous cell carcinoma	3/35 (8.6)	3/29 (10.3)	0 (0)
**Histological differentiation of primary tumor**			
Well	9/32 (28.1)	8/29 (27.6)	8/25 (32.0)
Moderate	8/32 (25.0)	7/29 (24.1)	6/25 (24.0)
Poor	15/32 (46.9)	14/29 (48.3)	11/25 (44.0)
Missing	3 (8.6)	0 (0)	0 (0)
**cT**			
cT1	0 (0)	0 (0)	0 (0)
cT2	1/34 (2.9)	1/28 (3.6)	1/24 (4.2)
cT3	21/34 (61.8)	16/28 (57.1)	14/24 (58.3)
cT4a	11/34 (32.4)	10/28 (35.7)	8/24 (33.3)
cT4b	1/34 (2.9)	1/28 (3.6)	1/24 (4.2)
Missing	1 (2.9)	1 (3.4)	1 (4.0)
**cN**			
cN0	6/34 (17.6)	5/28 (17.9)	4/24 (16.7)
cN1	9/34 (26.5)	7/28 (25.0)	7/24 (29.2)
cN2	10/34 (29.4)	7/28 (25.0)	7/24 (29.2)
cN3	9/34 (26.5)	9/28 (32.1)	6/24 (25.0)
Missing	1 (2.9)	1 (3.4)	1 (4.0)
**HER2 positive**	6/25 (24.0)	6/22 (27.3)	5/20 (25.0)
Missing	10 (28.6)	7 (24.1)	5 (20.0)
**Timing of detection**			
Synchronous	29/35 (82.9)	23/29 (79.3)	20/25 (80.0)
Metachronous	6/35 (17.1)	6/29 (20.7)	5/25 (20.0)
**Location of oligometastatic lesion**			
Liver metastases	16/35 (45.7)	11/29 (37.9)	11/25 (44.0)
Extra-regional lymph node metastases only	16/35 (45.7)	15/29 (51.7)	12/25 (48.0)
• Cervical or paratracheal M1 lymph nodes	3/35 (8.6)	3/29 (10.3)	3/25 (12.0)
• Thoracic M1 lymph nodes	1/35 (2.9)	1/29 (3.4)	0/25 (0)
• Abdominal M1 lymph nodes	10/35 (28.6)	9/29 (31.0)	8/25 (32.0)
• Multiple M1 lymph node regions	2/35 (5.7)	2/29 (6.9)	1/25 (4.0)
Liver and extra-regional lymph node metastases	3/35 (8.6)	3/29 (10.3)	2/25 (8.0)

### Baseline characteristics

In the overall cohort, including all 35 patients, the median age at diagnosis was 66 years, and 23 (65.7%) patients were male. Sixteen (45.7%) patients had metastases located only in the liver, another 16 (45.7%) patients had metastases in extra-regional lymph nodes, and 3 (8.6%) patients had metastases in both the liver and extra-regional lymph nodes. Patients unable to complete curative treatment (*n* = 6) were mainly men, elderly, had synchronous liver lesions, and had a poorly differentiated primary tumor. Baseline characteristics for the 29 patients who underwent curatively intended treatment (Completed Curative treatment) and the 25 patients with adenocarcinoma who underwent surgical resection or liver ablation (Curative resection Adenocarcinoma) are displayed in Table 1.

### Curatively intended treatment

Treatment for the 28 patients who underwent preoperative chemotherapy and curatively intended local treatment (i.e., surgical resection *n* = 27 or liver ablation *n* = 1) and for the patient who received definitive chemoradiotherapy is displayed stratified by oligometastatic location in Table 2 and Supplementary Information. Fifteen patients (53.6%) received fluorouracil, leucovorin, oxaliplatin, and docetaxel (FLOT), 8 (28.6%) patients received a combination of Oxaliplatin and 5-FU, and 5 (17.9%) were treated with other preoperative regimens. Preoperative chemotherapy was given at a reduced dose in 14 (58.3%) patients. Treatment interruption due to toxicity was required in 4 (14.3%) patients. In addition to chemotherapy, one (3.6%) patient received Trastuzumab and one (3.6%) patient received Nivolumab.

**Table 2:** Treatment stratified by site of oligometastatic disease

*n*/total (%)	Liver *n* = 11	Lymph node *n* = 15	Combined liver and lymph node *n* = 3
**Type of preoperative treatment**			
FLOT	5/11 (45.5)	9/14 (64.3)	1/3 (33.3)
Oxaliplatin + 5-FU	3/11 (27.3)	4/14 (28.6)	1/3 (33.3)
EOX	1/11 (9.1)	0 (0)	0 (0)
Irinotecan + 5-FU	1/11 (9.1)	0 (0)	0 (0)
Other or combination	1/11 (9.1)	1/14 (7.1)	1/3 (33.3)
**Preoperative treatment**			
Treatment interruption	4/11 (36.4)	0 (0)	0 (0)
Missing	0 (0)	0 (0)	0 (0)
Dose reduction	4/10 (40.0)	8/12 (66.7)	2/2 (100)
Missing	1 (9.1)	2 (14.3)	1 (33.3)
**Preoperative Nivolumab**	0 (0)	0 (0)	1/3 (33.3)
**Preoperative Trastuzumab**	0 (0)	0 (0)	1/3 (33.3)
**Treatment of the primary tumor**			
Esophagectomy	7/9 (77.8)	10/12 (83.3)	2/2 (100)
• Ivor Lewis	4/5 (80.0)	1/9 (11.1)	2/2 (100)
• McKeown	1/5 (20.0)	5/9 (55.6)	0 (0)
• Transhiatal esophagectomy	0 (0)	3/9 (33.3)	0 (0)
• Missing	2 (28.6)	1 (10.0)	0 (0)
Total gastrectomy	1/9 (11.1)	1/12 (8.3)	0 (0)
Subtotal gastrectomy	1/9 (11.1)	0 (0)	0 (0)
Definitive chemoradiotherapy	0 (0)	1/12 (8.3)	0 (0)
**Surgical approach**			
Open	3/9 (33.3)	5/13 (38.5)	2/3 (66.7)
Hybrid	3/9 (33.3)	4/13 (30.8)	1/3 (33.3)
Minimally invasive	3/9 (33.3)	4/13 (30.8)	0 (0)
Missing	2 (18.2)	1 (7.1)	0 (0)
**Local treatment of oligometastatic lesion**			
Liver resection	10/11 (90.9)	0 (0)	0 (0)
Liver ablation	1/11 (9.1)	0 (0)	0 (0)
Lymph node dissection*	0 (0)	14/15 (93.3)	0 (0)
Definitive chemoradiotherapy	0 (0)	1/15 (6.7)	0 (0)
Combined liver and lymph node dissection	0 (0)	0 (0)	3/3 (100)
**Adjuvant treatment**	6/10 (60.0)	5/11 (45.5)	1/3 (33.3)
Missing	1 (9.1)	3 (21.4)	0 (0)

* See Supplementary Information for details regarding lymph node stations

### Surgery

Surgical resection of the primary tumor with synchronous resection, or liver ablation, of the metastases was performed in 22 (78.6%) patients. Six (21.4%) patients were treated for metachronous metastases and underwent surgical resection of the metastases only. One (3.4%) patient received definitive chemoradiotherapy. Esophagectomy was performed in the majority of patients, 86.4%. Seven (43.8%) underwent transthoracic esophagectomy with a thoracic anastomosis, and 6 (37.5%) transthoracic esophagectomy with a cervical anastomosis. Total gastrectomy was performed in two (8.7%) patients, and subtotal gastrectomy in one (4.3%) patient. Open surgery was performed in 10 (40.0%) patients, hybrid in 8 (32.0%) patients, and minimally invasive surgery in 7 (28.0%) patients. Oligometastatic liver lesions (*n* = 11) were treated with liver resection in 10 (90.9%) patients and with liver ablation in one (9.1%) patient. All liver metastases were ≤ 30 mm. Nine (64.3%) patients had only one liver metastasis, 3 (21.4%) patients had two liver metastases, and 2 (14.3%) patients had three liver metastases. Regarding oligometastatic lymph node disease (*n* = 15), lymph node dissection was performed in 14 (93.3%) and definitive chemoradiotherapy was given in 1 (6.7%) patient. Combined liver and extra-regional lymph node resection was performed in all 3 (100%) patients with metastases in both sites. A description of resected lymph nodes is available in Supplementary Information. In summary, lymph node station 16 was the most common, resected in 11 (78.6%) patients, whereas cervical and thoracic lymph nodes were less common. Twelve (50.0%) patients received adjuvant chemotherapy.

### Postoperative complications

Postoperative complications occurred in 19 (73.1%) patients (Table 3). Three (11.5%) patients had complications of Clavien-Dindo grade I, 4 (15.4%) patients of grade II, 4 (15.4%) of grade IIIa, 4 (15.4%) of grade IIIb, and 4 (15.4%) patients had complications of grade IVa. The 4 patients with grade IVa complications were all treated for synchronous oligometastatic disease, one with liver metastases and three with extra-regional lymph node metastases. All patients underwent esophagectomy, one with open surgery and three with minimally invasive technique. The first patient was admitted to the intensive care unit (ICU) for three days due to delayed extubation after a long operation and developed recurrent laryngeal nerve palsy. The second patient was reintubated in the operating room and transferred to the ICU for three days due to hemodynamic instability and suspected systemic inflammatory response syndrome (SIRS). The third patient developed an anastomotic leak that was treated endoscopically and was admitted to the ICU seven days after surgery due to respiratory failure and sepsis. This patient stayed at the ICU for one day. The fourth patient developed respiratory failure and sepsis postoperatively and stayed at the ICU for 13 days for respiratory support and received a tracheostomy. All four patients recovered and were discharged from the hospital.

Anastomotic leak was observed in 3 (11.5%) patients. One patient had a minor leak and was treated conservatively with antibiotics. Two patients were treated endoscopically. Infection was observed in 9 (34.6%) patients. Two (7.7%) patients had sepsis, 4 (15.4%) had wound infections, and the remaining 3 (11.5%) patients had postoperative infections of unknown origin or other locations. Two (7.7%) patients had recurrent laryngeal nerve palsy. Median hospital stay was 12.5 days (IQR 6.75-21.0), and median ICU stay was 3 days (IQR 2.5–5.5).

### Postoperative mortality

No patient died within the first 30 days of surgery, but one patient died within 90 days (3.4%) due to early tumor recurrence. This patient underwent esophagectomy with synchronous lymphadenectomy, but tumor was found at the circumferential resection margin. The patient was readmitted to the hospital shortly after discharge, and a CT scan revealed malignant thoracic lymph nodes.

### Treatment response

Residual tumor after preoperative treatment was > 50% in most patients, 12 (63.2%), 10–50% in 6 (31.6%) patients, and < 10% in one patient (11%). Tumor-free resection margins were achieved in 12 (60.0%) patients for the primary tumor and in 23 (88.5%) patients for metastases (Table 3). Non-radical circumferential resection margins were the most common, whereas tumor-free proximal and distal resection margins were achieved in 19 (95%) patients.

**Table 3:** Treatment outcome stratified by site of oligometastatic disease

*n*/total (%)	Liver *n* = 11	Lymph node *n* = 14	Combined liver and lymph node *n* = 3
**Toxicity**			
No toxicity	1/10 (10.0)	0 (0)	0 (0)
Grade 1–2	3/10 (10.0)	9/11 (81.8)	0 (0)
Grade 3–4	6/10 (60.0)	2/11 (18.2)	1/1 (100)
Missing	1 (9.1)	3 (21.4)	2 (66.7)
**Extent of resection: primary tumor**			
Tumor free resection margins	7/8 (87.5)	4/10 (40.0)	1/2 (50.0)
Tumor free proximal resection margin	8/8 (100)	9/10 (90.0)	2/2 (100)
Tumor free distal resection margin	8/8 (100)	9/10 (90.0)	2/2 (100)
Tumor free circumferential resection margin	7/8 (87.5)	4/10 (40.0)	1/2 (50.0)
Missing	1 (11.1)	1 (10.0)	0 (0)
**Extent of resection: metastases**			
Tumor free resection margins	10/10 (100)	11/13 (84.6)	2/3 (66.7)
Missing	1 (9.1)	1 (7.1)	0 (0)
**Tumor Regression Grade**			
No residual tumor	0 (0)	0 (0)	0 (0)
< 10% residual tumor	1/7 (14.3)	0 (0)	0 (0)
10–50% residual tumor	2/7 (28.6)	3/9 (33.3)	1/3 (33.3)
> 50% residual tumor	4/7 (57.1)	6/9 (66.7)	2/3 (66.7)
Missing	4 (36.4)	5 (35.7)	0 (0)
**Type of complication**			
Infection	4/10 (40.0)	4/13 (30.8)	1/3 (33.3)
Sepsis	1/10 (10.0)	1/13 (7.7)	0 (0)
Anastomotic leak	1/10 (10.0)	2/13 (15.4)	0 (0)
Respiratory complications	1/10 (10.0)	7/13 (53.8)	1/3 (33.3)
Other	2/10 (20.0)	2/13 (15.4)	1/3 (33.3)
Missing	1 (9.1)	1 (7.1)	0 (0)
**Clavien-Dindo grade**	7/10 (70.0)	10/13 (76.9)	2/3 (66.7)
No complication	3/10 (30.0)	3/13 (23.1)	1/3 (33.3)
I	2/10 (20.0)	1/13 (7.7)	0 (0)
II	2/10 (20.0)	1/13 (7.7)	1/3 (33.3)
IIIa	1/10 (10.0)	2/13 (15.4)	1/3 (33.3)
IIIb	1/10 (10.0)	3/13 (23.1)	0 (0)
IVa	1/10 (10.0)	3/13 (23.1)	0 (0)
IVb	0 (0)	0 (0)	0 (0)
V	0 (0)	0 (0)	0 (0)
Missing	1 (9.1)	1 (7.1)	0 (0)
**Length of hospital stay**			
Median hospital stay (IQR), days	11.0 (7.0–21)	14.0 (10.5–18.8)	8.0 (7.0-15.5)
Median ICU stay (IQR), days	13.0 (0)	3.0 (2.0–3.0)	N/A

### Survival after curative treatment

Median overall survival after curatively intended treatment in the entire group including both adenocarcinoma (*n* = 26) and squamous cell carcinoma (*n* = 3), as well as surgery (*n* = 28) and definitive chemoradiotherapy (*n* = 1) was 23.9 months (IQR 12.3–43.2) from diagnosis of oligometastatic disease and 18.4 months (IQR 7.7–38.6) from surgery or start of definitive chemoradiotherapy. The three patients with squamous cell carcinoma and the patient who received definitive chemoradiotherapy were excluded from all the following analyses.

### Survival after surgery in patients with adenocarcinoma

**Fig. 2:**
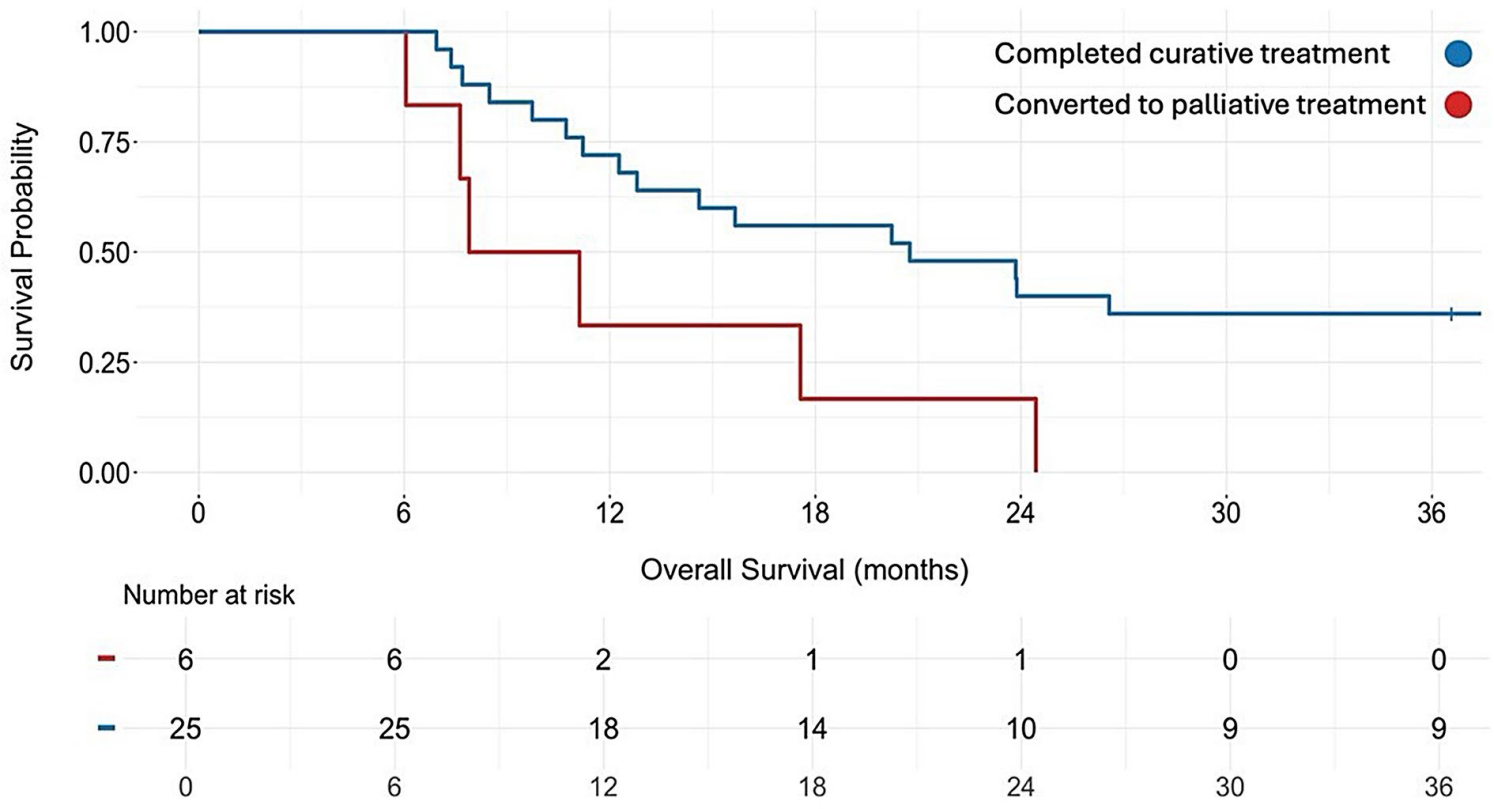
Overall survival for patients with gastroesophageal adenocarcinoma from diagnosis of oligometastatic disease stratified by treatment intent

Median overall survival in patients with adenocarcinoma who received neoadjuvant chemotherapy followed by surgery (*n* = 25) was 20.8 months (IQR 11.2–42.6) from diagnosis and 17.0 months (IQR 6.4–35.9) from surgery (Fig. 2).

Median overall survival in patients with adenocarcinoma who received FLOT followed by surgery (*n* = 14) was 22.0 months (IQR 11.6–43.1) from diagnosis, compared to 13.4 months (IQR 9.4–19.2) in patients who received Oxaliplatin/5-FU (*n* = 6). When stratified by oligometastatic location, median overall survival was 23.9 months (IQR 15.1–46.7) from diagnosis in patients with liver metastases (*n* = 11), and 12.0 months (IQR 8.3–30.1) from diagnosis in patients with extra-regional lymph node metastases (*n* = 12). Similarly, median overall survival was 17.9 months (IQR 10.8–41.0) in patients treated for the synchronous oligometastatic disease (*n* = 10) and 26.6 months (IQR 20.8–48.7) in patients treated for metachronous oligometastatic disease (*n* = 5). One-, two-, and three-year survival was 72.0%, 40.0%, and 33.3% from diagnosis. The subgroup of patients who survived at least three years had a higher proportion of females (44.4% vs. 32.0%), gastric cancer (33.3% vs. 12.0%), well-differentiated tumors (55.6% vs. 32.0%), and tumor-free resection margins (88.9% vs. 64.0%) compared to the total group of patients with adenocarcinoma. A poorly differentiated tumor was correlated to shorter survival (Table 4). No other significant correlation was found.

Median overall survival in the 6 patients who progressed during preoperative treatment and received palliative treatment was 9.5 months (IQR 7.7–16.0) from diagnosis.

### Progression-free survival

**Fig. 3:**
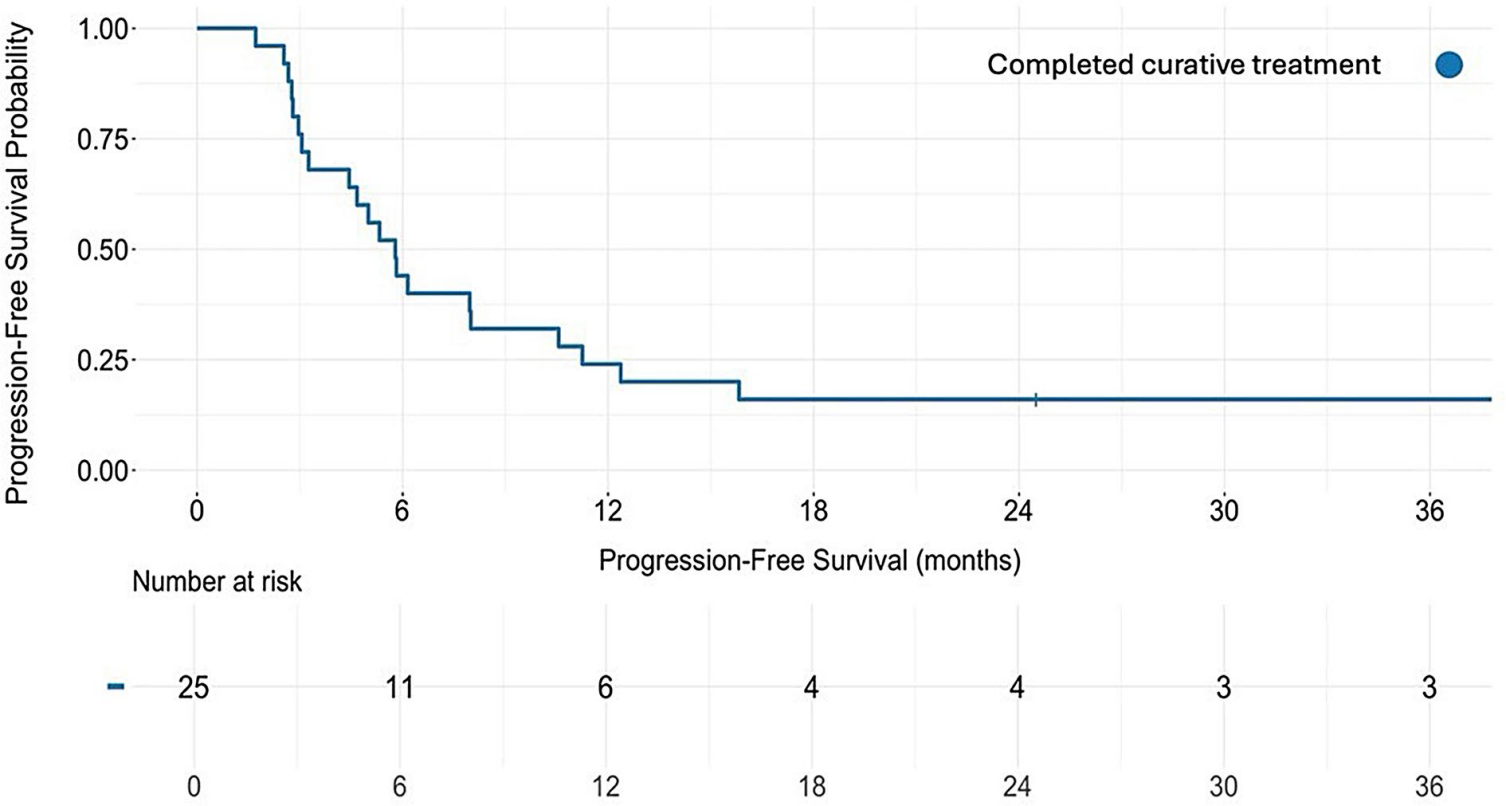
Progression-free survival for patients with gastroesophageal adenocarcinoma from surgery

Median progression-free survival in patients with adenocarcinoma who received neoadjuvant chemotherapy followed by surgery was 5.8 months (IQR 3.1–11.3) (Fig. 3). Median progression-free survival was 5.0 months (IQR 3.7–10.9) in patients with liver metastases and 5.7 months (IQR 3.0-13.2) in patients with extra-regional lymph node metastases.

**Table 4:** Hazard ratios for subgroups with 95% confidence intervals

	Hazard ratio	Confidence interval	*p*-value
**Sex**	0.60	0.21–1.69	0.33
Female (vs. male)			
**Age**	0.62	0.25–1.55	0.30
≥ 65 years at diagnosis (vs. <65 years at diagnosis)			
**Histological differentiation**	2.76	1.07–7.09	0.035*
Poor (vs. well-moderate)			
**Location of metastasis**	2.07	0.78–5.47	0.14
Lymph node (vs. liver)			
**Tumor Regression Grade**	0.69	0.23–2.05	0.50
≥ 50% residual tumor (vs. <50% residual tumor)			
**Timing of detection**	0.58	0.17–2.01	0.40
Metachronous (vs. synchronous)			
**Clavien-Dindo grade**	2.42	0.80–7.37	0.12
IIIb-V (vs. 0-IIIa)			
**Neoadjuvant chemotherapy**	1.71	0.57–5.17	0.30
Oxaliplatin-5-FU (vs. FLOT)			

### Quality of life

Results from QLQ-C30 and QLQ-OG25 at 3 and 12 months are displayed in Table 5. Response rates were overall low, 27.9% at 3 months and 10.3% at 12 months. At 3 months, social function received the lowest score in the functional subscale, and anxiety and dyspnea received the highest scores in the symptom subscale. At 12 months, there was an overall improvement, but role function, appetite loss, anxiety, and problems with dry mouth showed worse results.

**Table 5:** Patient-reported quality of life at 3 and 12 months after surgery

EORTC QLQ-C30	3 months		12 months	
	*n* = 10		*n* = 3	
**Functional subscale**	Mean	SD	Mean	SD
Physical function	80.0	19.6	88.9	10.2
Role function	78.3	32.4	61.1	34.7
Emotional function	83.3	15.2	88.9	19.2
Cognitive function	91.7	11.8	100.0	0.0
Social function	61.7	26.1	66.7	33.3
**Symptom subscale**				
Fatigue	38.9	21.1	29.6	28.0
Nausea/vomiting	11.7	11.2	11.1	9.6
Pain	26.7	23.8	5.6	9.6
Dyspnea	50.0	23.6	22.2	19.2
Insomnia	20.0	23.3	0.0	0.0
Appetite loss	13.3	17.2	33.3	33.3
Constipation	10.0	31.6	0.0	0.0
Diarrhea	26.7	30.6	11.1	19.2
Financial difficulties	0.0	0.0	0.0	0.0
**Global scale**	66.7	21.9	72.2	34.7
**QLQ-OG25**	*n* = 10		*n* = 3	
	Mean	SD	Mean	SD
Dysphagia	12.2	25.4	0.0	0.0
Eating	28.3	29.2	22.2	21.0
Reflux	8.3	14.2	5.6	9.6
Odynophagia	16.7	17.6	0.0	0.0
Pain and discomfort	21.7	15.8	11.1	19.2
Anxiety	48.3	33.7	50.0	28.9
Eating with others	3.3	10.5	0.0	0.0
Dry mouth	20.0	28.1	33.3	33.3
Trouble with taste	33.3	38.5	22.2	38.5
Body image	20.0	23.3	11.1	19.2
Trouble swallowing saliva	6.7	14.5	11.1	19.2
Choked when swallowing	3.3	10.5	0.0	0.0
Trouble with coughing	33.3	38.5	0.0	0.0
Trouble talking	10.0	16.1	0.0	0.0
Weight loss	36.7	33.1	22.2	19.2
Hair loss	41.7	41.9	16.7	23.6

## Discussion

This prospective multicenter study assessed the safety and feasibility of curative treatment in 29 patients with oligometastatic gastroesophageal cancer. We found the rate of postoperative complications to be relatively high but not associated with postoperative mortality.

Treatment safety is a crucial aspect in the discussion of a more extensive treatment procedure. Previous clinical trials of curative treatment for locally advanced gastroesophageal cancer have reported postoperative complications in about 22–50% of patients and postoperative mortality in 2–6% [29–31]. Two more recent registry-based studies found postoperative complications after esophagectomy in 59% and 76% of patients and complications of Clavien-Dindo grade IIIb or higher in 17.2% [32, 33]. To our knowledge, only a few studies have described postoperative complications and mortality in oligometastatic disease with rates between 19% and 59% [13, 15, 23, 24, 34, 35]. A German cohort study including 48 patients with oligometastatic gastroesophageal cancer found an overall complication rate of 48%, with 59% after esophagectomy and 42% after gastrectomy [13]. Similarly, a Chinese registry-based study reported complications in 48% of patients after gastrectomy with synchronous or metachronous liver metastases [34], and another study found complications to be similar to the rate after resection of the primary tumor alone [23]. Two studies found complications in 55% and 19% of patients after gastrectomy or esophagectomy with simultaneous resection or ablation of liver metastases, with the latter study mostly including patients with oligometastatic gastric cancer [15, 35]. Postoperative mortality has been reported in 0–6% after surgery for oligometastatic gastroesophageal cancer [13, 34, 35].

Twenty-nine of 35 patients were able to complete curative treatment in the present study. The overall complication rate was higher compared with previous studies on oligometastatic disease but similar to what has been reported after esophagectomy in non-metastatic disease. However, postoperative mortality was similar to previous rates in both oligometastatic and non-metastatic disease. One possible reason for the higher rate of complications might be the higher proportion of esophagectomies in the present study compared with previous studies on oligometastatic disease. How complications have been reported could also have had an impact on the results.

Overall survival in patients with gastroesophageal adenocarcinoma in the present study was 20.8 months from diagnosis of oligometastatic disease. This could be compared with 9.5–11 months after first-line palliative chemotherapy or the slightly longer survival with the addition of targeted treatments [36–38]. However, only 2 (6.9%) patients received preoperative Trastuzumab or Nivolumab, and the present study may not be representative of this group [39–41]. Previous studies on oligometastatic gastroesophageal cancer, especially after the introduction of perioperative chemotherapy, have shown varying results after curatively intended treatment with a median overall survival between 9.3 and 35 months [13–16, 24, 42, 43]. For instance, in the AIO-FLOT3 Trial, patients with limited metastatic gastric or gastroesophageal junction cancer who underwent systemic treatment and subsequent resection had a median overall survival of 31.3 months [44]. A subsequent registry-based study reported a median overall survival of 16.7 months after metastasectomy for oligometastatic gastroesophageal cancer [15]. Similarly, a recent multicenter study found overall survival to be 35 months in 27 patients with oligometastatic gastroesophageal cancer who underwent combined systemic and local treatment (i.e., metastasectomy, definitive chemoradiotherapy, or stereotactic body radiation therapy) [12]. In contrast, other previous studies did not find a survival benefit after surgery in oligometastatic gastroesophageal cancer [13, 45]. Metastases were generally not resected in the REGATTA trial, which differs from many of the later studies, including the present study.

Overall survival in the present study was comparable to previous studies on oligometastatic disease but towards the lower range. A subgroup of the included patients seemed to respond better to the treatment and had longer overall survival, whereas others developed an early recurrence. The included patients represent a quite heterogeneous group with different metastatic locations, timing of metastatic disease, and given treatment. Therefore, the different survival rates based on chemotherapy regimen and metastatic location should be interpreted cautiously, as there might be baseline differences that were not included in the analysis. Besides histological differentiation grade, we found no correlation between patient or tumor characteristics and survival, but the small sample size could have an impact on these results.

The variation in survival among previous studies on oligometastatic gastroesophageal cancer could, to some degree, be related to differences in study design and small patient groups, including the definition of oligometastatic disease used in previous studies. The present study also used a slightly wider definition of oligometastatic disease, including the three patients with combined liver and extra-regional lymph node metastases. The recent RENAISSANCE phase III trial included a larger cohort and was designed to evaluate the addition of surgical resection in oligometastatic gastric and gastroesophageal junction cancer but did unfortunately not meet the primary endpoint [46].

Limitations of the current study include the non-randomized design, heterogeneous patient group, limited sample size, and missing data concerning treatment and postoperative complications in some patients. The fact that baseline differences were not adjusted for in the survival analysis, including different chemotherapy regimens, may impact the survival. All these factors should be considered when interpreting the results. The absence of a validated international definition of oligometastatic gastroesophageal cancer limits the study’s external validity. Strengths include the complete and detailed follow-up concerning survival, and the prospective study design.

## Conclusion

This study shows that curatively intended treatment could be completed in the majority of patients with an overall survival of 20.8 months (IQR 11.2–42.6). Postoperative complications were common, but not related to postoperative mortality. Future randomized studies including patients with a clear definition of oligometastatic disease are warranted to assess the utility of resection in oligometastatic gastroesophageal cancer, and identify factors associated with favorable outcomes.

## Electronic supplementary material

Below is the link to the electronic supplementary material.

Supplementary Material 1

## Data Availability

Data is provided within the manuscript and supplementary information. A complete dataset is stored at Karolinska Institutet. The dataset will only be available for relevant questions and as a control for the data in the manuscript.
