# Supplementary material for: Curative treatment for oligometastatic gastroesophageal cancer– results of a prospective multicenter study
Source: Langenbecks Arch Surg. 2024 Dec 16;410(1):10. doi: 10.1007/s00423-024-03575-7 (PMC11649779; doi:10.1007/s00423-024-03575-7)
Supplement: Supplementary file 1 — Supplementary Material 1 [file 423_2024_3575_MOESM1_ESM.docx]

| **Patient ID** | **Neoadjuvant treatment** | **Dose** | **Cycles** | **Interruption** | **Toxicity** | **Resection** | **Adjuvant treatment** | **Dose** | **Cycles** | **Interruption** | **Toxicity** |
| --- | --- | --- | --- | --- | --- | --- | --- | --- | --- | --- | --- |
| **Liver metastases: completed curative treatment** | | | | | | | | | | | |
| 2 | EOX | 100% | 3 | No | Nausea, diarrhea  CTCAE 1 | Subtotal gastrectomy  + liver resection | EOX | <100% | 3 | No | Peripheral neuropathy  CTCAE 1 |
| 4 | Oxaliplatin-5-FU | 100% | 6 | No | Minor stroke CTCAE 4 | Esophagectomy  + liver resection | No | N/A | N/A | N/A | N/A |
| 16 | FLOT | 100% | 3 | Yes | Colitis  CTCAE 3 | Esophagectomy  + liver resection | No | N/A | N/A | N/A | N/A |
| 20 | Oxaliplatin-5-FU | <100% | 4 | No | Pain  CTCAE 1 | Esophagectomy  + liver resection | Oxaliplatin-5-FU Trastuzumab | <100% | 4 | No | Nausea, diarrhea  CTCAE 2 |
| 21 | Oxaliplatin-5-FU | 100% | 5 | Yes | Hypokalemia, diarrhea, affected hearing  CTCAE 3 | Liver resection | Oxaliplatin-5-FU | 100% | 3 | Yes | Fatigue, diarrhea  CTCAE 2 |
| 22 | Oxaliplatin-Docetaxel | 100% | 4 | No | Peripheral neuropathy.  CTCAE 2 | Esophagectomy  + liver resection | No | N/A | N/A | N/A | N/A |
| 24 | FLOT | <100% | 4 | No | Peripheral neuropathy, diarrhea, febrile neutropenia  CTCAE 3 | Total gastrectomy  + liver resection | Oxaliplatin-5-FU | <100% | 4 | No | Peripheral neuropathy, diarrhea  CTCAE 1 |
| 31 | Irinotecan-5-FU | <100% | 5 | Yes | Nausea, infection  CTCAE 3 | Liver resection | No | N/A | N/A | N/A | N/A |
| 35 | FLOT | <100% | 4 | No | Missing | Esophagectomy  + liver ablation | Oxaliplatin-5-FU | <100% | 3 | Missing | Peripheral neuropathy, nausea |
| 36 | FLOT | Missing | 1 | Yes | Neutropenia, intestinal hemorrhage  CTCAE 3 | Esophagectomy  + liver resection | Missing | Missing | Missing | Missing | Missing |
| 38 | FLOT | 100% | 4 | No | No | Esophagectomy  + liver resection | FLOT | 100% | 4 | No | Nausea, fatigue  CTCAE 1 |
| **Liver metastases: converted to palliative treatment** | | | | | | | | | | | |
| 3 | Irinotecan-5-FU | <100% | 4 | No | Fatigue  CTCAE 1 | Progression during preoperative chemotherapy | N/A | N/A | N/A | N/A | N/A |
| 9 | Oxaliplatin-5-FU | 100% | 5 | No | No | Progression during preoperative chemotherapy | N/A | N/A | N/A | N/A | N/A |
| 12 | FLOT | 100% | 4 | No | Sepsis  CTCAE 3 | Progression during preoperative chemotherapy | N/A | N/A | N/A | N/A | N/A |
| 15 | FLOT | 100% | 4 | No | No | Progression during preoperative chemotherapy | N/A | N/A | N/A | N/A | N/A |
| 29 | FLOT | Missing | 6 | No | Missing | Progression during preoperative chemotherapy | N/A | N/A | N/A | N/A | N/A |
| **Lymph node metastases: completed curative treatment** | | | | | | | | | | | |
| 5 | Oxaliplatin-5-FU | <100% | 5 | No | Peripheral neuropathy, mucositis, diarrhea  CTCAE 2 | Esophagectomy  + lymphadenectomy | No | N/A | N/A | N/A | N/A |
| 6 |  |  |  |  |  | Definitive chemoradiotherapy | No | N/A | N/A | N/A | N/A |
| 7 | Oxaliplatin-5-FU | <100% | 3 | No | Nausea, mucositis CTCAE 3 | Esophagectomy  + lymphadenectomy  #16a, 16b, 101 | No | N/A | N/A | N/A | N/A |
| 8 | FLOT | <100% | 4 | No | Constipation, abdominal pain  CTCAE 3 | Esophagectomy  + lymphadenectomy  #104 | No | N/A | N/A | N/A | N/A |
| 11 | Oxaliplatin-5-FU | 100% | 3 | No | Nausea  CTCAE 1 | Lymphadenectomy | Oxaliplatin-5-FU  RT | 100% | 3 | No | Nausea  CTCAE 1 |
| 13 | FLOT  Carboplatin-Docetaxel | 100% | 4 | No | Nausea, acute toxic reaction  CTCAE 2 | Lymphadenectomy  #16a, 16b | CROSS | Missing | 5 | No | Nausea, abdominal pain  CTCAE 1 |
| 14 | FLOT | <100% | 4 | No | Diarrhea, abdominal pain  CTCAE 1 | Esophagectomy  + lymphadenectomy  #16b | No | N/A | N/A | N/A | N/A |
| 17 | FLOT | <100% | 4 | No | Peripheral neuropathy  CTCAE 1 | Lymphadenectomy  #101, 104 | RT | N/A | N/A | No | Skin reaction  CTCAE 1 |
| 19 | Oxaliplatin-5-FU | <100% | 7 | No | Mucositis  CTCAE 1 | Esophagectomy  + lymphadenectomy  #106 | No | N/A | N/A | N/A | N/A |
| 25 | FLOT | 100% | 4 | No | Peripheral neuropathy  CTCAE 1 | Esophagectomy  + lymphadenectomy  #16b, 101 | FLOT | Missing | 3 | Yes | Hypokalemia, diarrhea  CTCAE 1 |
| 26 | FLOT | <100% | 4 | No | Peripheral neuropathy, abdominal pain, diarrhea  CTCAE 2 | Esophagectomy  + lymphadenectomy  #16a, 16b | No | N/A | N/A | N/A | N/A |
| 28 | FLOT | 100% | 4 | No | Diarrhea  CTCAE 1 | Esophagectomy  + lymphadenectomy  #16b | FLOT | <100% | 4 | No | Diarrhea  CTCAE 1 |
| 30 | FLOT | Missing | 4 | No | Missing | Esophagectomy  + lymphadenectomy  #16a, 16b | Missing | Missing | Missing | Missing | Missing |
| 34 | FLOT | Missing | 4 | No | Missing | Esophagectomy  + lymphadenectomy | Missing | Missing | Missing | Missing | Missing |
| 37 | FLOT | <100% | 4 | No | Missing | Total gastrectomy  + lymphadenectomy  #16a, 16b | Missing | Missing | Missing | Missing | Missing |
| **Lymph node metastases: converted to palliative treatment** | | | | | | | | | | | |
| 18 | FLOT | Missing | 4 | No | Missing | Progression during preoperative chemotherapy | N/A | N/A | N/A | N/A | N/A |
| **Combined lymph node and liver metastases: completed curative treatment** | | | | | | | | | | | |
| 1 | Oxaliplatin-5-FU Trastuzumab | Missing | Missing | No | Missing | Liver resection  + lymphadenectomy  #16a, 16b | No | N/A | N/A | N/A | N/A |
| 27 | FLOT  Irinotecan-5-FU Nivolumab | <100% | 4 | No | Febrile neutropenia,  abdominal pain  CTCAE 3 | Esophagectomy  + lymphadenectomy  #16a  + liver resection | No | N/A | N/A | N/A | N/A |
| 33 | FLOT | <100% | 4 | No | Diarrhea, laryngospasm  CTCAE missing | Esophagectomy  + lymphadenectomy  #16b  + liver resection | Oxaliplatin-5-FU | <100% | 3 | No | Diarrhea  CTCAE 1 |
| Abbreviation: CTCAE = Common Terminology Criteria for Adverse Events, FLOT = fluorouracil, leucovorin, oxaliplatin and docetaxel, EOX = epirubicin, capecitabine and oxaliplatin, 5-FU = 5-fluorouracil, RT = radiotherapy. | | | | | | | | | | | |
|  |  |  |  |  |  |  |  |  |  |  |  |
